# Reactions to vaping cessation messaging and strategies among US young adults who use e-cigarettes

**DOI:** 10.18332/tpc/186271

**Published:** 2024-04-11

**Authors:** Katelyn F. Romm, Daisy Le, Lorien C. Abroms, Carla J. Berg

**Affiliations:** 1 Health Promotion Research Center, Stephenson Cancer Center, University of Oklahoma Health Sciences Center, Oklahoma City, United States; 2 Department of Pediatrics, College of Medicine, University of Oklahoma Health Sciences Center, Oklahoma City, United States; 3 Department of Policy, Populations, and Systems, School of Nursing, George Washington University, Washington, United States; 4 Department of Prevention and Community Health, Milken Institute School of Public Health, George Washington University, Washington, United States; 5 George Washington Cancer Center, George Washington University, Washington, United States

**Keywords:** e-cigarette use, young adults, tobacco cessation, cigarette use, cannabis use

## Abstract

**INTRODUCTION:**

Despite high rates of young adult e-cigarette use, research informing cessation interventions for this population, including those unready to quit, is limited.

**METHODS:**

We analyzed 2022 cross-sectional survey data (from a larger longitudinal study) among 172 US young adult, past-month e-cigarette users (mean age=26.95 years; 57.6% female; 73.3% White). We examined: 1) perceived challenges to quitting e-cigarettes; 2) perceived impact of intervention messages targeting motivation and confidence, and their associations with quitting importance and confidence; and 3) poly-use with cigarettes and/or cannabis in relation to poly-product cessation preferences.

**RESULTS:**

In all, 43.6% reported past-year e-cigarette quit attempts, and 55.3% reported quit readiness; 30.2% reported past-month poly-use with cigarettes, and 54.1% with cannabis. Frequently endorsed challenges to quitting/cutting down were related to stress/anxiety (41.9%), delaying cessation attempts (35.5%), and boredom (25.6%). Messages targeting motivation perceived as most impactful (scale: 1 = ‘no impact at all’ to 7 = ‘a lot of impact’) were related to saving money (mean score=4.78), improving health (mean score=4.15), and avoiding toxic chemicals (mean score=4.04), messages targeting confidence perceived as most impactful were related to patience (mean score=4.47), staying busy (mean score=4.27), and soliciting support (mean score=3.84). Perceiving greater impact of messages targeting motivation was associated with greater quitting importance (B=1.16; 95% CI: 0.71–1.60, p<0.001). Neither perceived impact of messages targeting motivation nor confidence were related to quitting confidence. E-cigarette–cannabis poly-users preferred to quit cigarettes first, e-cigarette–cigarette users preferred to quit cannabis first, and poly-users of all three products preferred to quit e-cigarettes first, followed by cigarettes, then cannabis.

**CONCLUSIONS:**

E-cigarette cessation interventions must use relevant messages (e.g. target financial and health benefits of quitting) and consider poly-users’ preferences for quitting.

## INTRODUCTION

The past decade has involved increases in e-cigarette use, particularly among young adults^1^. Compared to other age groups, young adults report higher use prevalence^1^, which has nearly doubled from 5.2% (2014) to 9.3% (2019)^1^. Although e-cigarettes may provide a less harmful alternative for cigarette smokers^2^, they pose significant health risks if sustained (e.g. the uptake of cigarettes, nicotine dependence)^3^. Thus, research to inform the development of accessible and effective e-cigarette cessation interventions among young adults is critical.

Young adults report a greater preference for technology-based cessation interventions (particularly those using text messaging) over in-person/phone-based counseling^4^. Mobile health (mHealth) text messaging interventions are an effective strategy for cigarette cessation, with reach across subpopulations (e.g. racial/ethnic, socioeconomic groups)^5^. There is a dearth of research on the effectiveness of mHealth interventions for e-cigarette use cessation, particularly for young adults, with only one study to our knowledge, demonstrating the effectiveness of a text messaging intervention on e-cigarette cessation among young adults^6^.

E-cigarette cessation interventions must address the range of factors that influence e-cigarette use. Social Cognitive Theory (SCT) highlights the importance of accounting for the cognitive, behavioral, and socioenvironmental influences (e.g. harm perceptions, use profiles, cravings/triggers, social support) on e-cigarette use and cessation^7^. Notably, existing e-cigarette cessation interventions have not incorporated a broad range of young adults, including those who are not ready to quit e-cigarette use. Moreover, research is lacking regarding differences in cessation preferences based on e-cigarette use profiles, including the use of e-cigarettes along with other products, such as cigarettes and cannabis (which are highly comorbid with e-cigarette use among young adults)^8,9^.

Informed by SCT, this study examined: 1) young adults’ perceived challenges to quitting/cutting down e-cigarette use; 2) perceived impact of intervention messages targeting motivation and confidence to quit, respectively; 3) associations between perceived impact of e-cigarette intervention messages targeting importance and confidence in relation to self-reported e-cigarette quit importance and confidence; and 4) associations among e-cigarette use profiles (i.e. poly-use with cigarettes and/or cannabis) on preferred strategies for quitting e-cigarettes, cigarettes, and cannabis among a sample of young adults who report past-month e-cigarette use.

## METHODS

### Study design

This study analyzed cross-sectional data from the Vape shop Advertising, Place characteristics, and Effects Surveillance (VAPES) longitudinal study^10^, which included young adults from 6 metropolitan statistical areas (MSAs; Atlanta, Boston, Minneapolis, Oklahoma City, San Diego, Seattle) across the US with varied tobacco legislation^11^. Participants (i.e. aged 18–34 years, residing in one of the 6 MSAs, English-speaking) were recruited in Fall 2018 via targeted Facebook and Reddit ads and were directed to an online consent form and eligibility screener. Eligible individuals were routed to complete the online baseline survey. Purposive, quota-based sampling ensured sufficient numbers of cigarette/e-cigarette users (about 1/3 each), men/women, and racial/ethnic minorities.

Of the 10433 individuals who clicked ads, 9847 consented, of which 2751 were not allowed to advance (i.e. ineligible and/or excluded from reaching subgroup target enrollment). Of those who advanced, 3006 completed the survey and enrollment^10^. Additional study details are elsewhere^10^. The current study analyzed Spring 2022 data from a subset of participants, selected based on sociodemographics and tobacco use. Of 1147 invited, 942 (82.1%) participated (compensated via $10 e-gift cards). Analyses focus on 172 participants who reported past-month (current) e-cigarette use.

### Measures

*E-cigarette, cigarette, and cannabis use*

We assessed a number of days of e-cigarette, cigarette, and cannabis use in the past 30 days. We also assessed e-cigarette use characteristics, including how often participants typically use e-liquids with THC and how often they typically use e-liquids with nicotine salt (never, rarely, some of the time, most of the time, all of the time). Those who reported some, most, or all of the time were classified as typically using e-liquids with THC and nicotine salt, respectively. Participants indicated the type of e-cigarette device they use most often (disposable device, device with replaceable prefilled cartridges, device with a tank that refills with liquids, mod system) and the 3 flavors they use most often (tobacco, menthol, fruit/dessert, other).

*E-cigarette cessation-related factors*

Participants indicated the importance of quitting and their confidence in quitting e-cigarettes (0 = ‘not at all’ to 10 = ‘absolutely’). They also reported past 12-month e-cigarette quit attempts (i.e. number of times they stopped using e-cigarettes for ≥1 days because they were trying to quit; 0–31+) and readiness to quit e-cigarettes (i.e. seriously thinking about quitting: yes, within the next 30 days; yes, within the next 6 months; yes, in more than 6 months; not thinking about quitting).

*E-cigarette cessation/reduction challenges*

A list of 13 common challenges to e-cigarette cessation was developed based on the literature^12^; participants indicated whether they had ever experienced each when trying to quit or cut down e-cigarette use (e.g. ‘I struggled with withdrawal symptoms’, ‘People important to me made it a challenge to resist vaping’, ‘I told myself vaping doesn’t pose any real health risks’).

*Impact of e-cigarette cessation messages on motivation and confidence*

Participants were asked to indicate the impact of 5 distinct messages targeting motivation (e.g. ‘Did you know that over 60% of young adults like you want to quit vaping?’) and the impact of 8 distinct messages targeting confidence (e.g. ‘Remember that quitting is a process; be patient with yourself and proud of all your hard work’) on their quitting motivation and confidence, respectively (1 = ‘no impact at all’ to 7 = ‘a lot of impact’). For primary analyses, items were summarized as the average response for the perceived impact of messages targeting motivation (correlations >0.4, p<0.001; Cronbach’s alpha=0.85) and messages targeting confidence (correlations >0.4, p<0.001; Cronbach’s alpha=0.90), respectively.

*Recommendations for quitting poly-use of e-cigarettes, cigarettes, and cannabis*

Participants responded to 2 items asking: ‘How would you recommend that someone quit smoking cigarettes and vaping?’ (quit both at the same time; quit smoking cigarettes first, then quit vaping; quit vaping first, then quit smoking cigarettes) and ‘How would you recommend that someone quit smoking cigarettes and vaping and using marijuana?’ (quit all three at the same time; quit smoking cigarettes first, then quit using marijuana second, then quit vaping nicotine; quit using marijuana first, then quit smoking cigarettes second, then quit vaping nicotine; quit smoking cigarettes first, then quit vaping nicotine second, then quit using marijuana; quit vaping nicotine first, then quit smoking cigarettes, then quit using marijuana).

*Sociodemographic characteristics*

We coded participants’ MSA of residence and assessed their age, sex, race, ethnicity, and sexual orientation.

### Statistical analysis

Descriptive statistics characterized the sample. Multivariable linear regressions examined participants’ perceived impact of intervention messages targeting motivation and confidence in relation to self-reported quit importance and quit confidence, respectively, controlling for sociodemographics [i.e. age, sex (ref. male), race (ref. White), ethnicity (ref. non-Hispanic), sexual orientation (ref. heterosexual)], number of days of past-month e-cigarette use, and other substance use (i.e. any past-month cigarette use and cannabis use). Chi-squared analyses examined associations between participants’ poly-use status (no poly-use, e-cigarette-cigarette use, e-cigarette-cannabis use, use of all three) and recommendations for quitting the use of e-cigarettes and cigarettes, as well as quitting the use of e-cigarettes, cigarettes, and cannabis. Analyses were conducted using SPSS v26. There were no missing data on any key study variables.

## RESULTS

In this sample of young adult e-cigarette users [mean age=26.95 years; 57.6% female, 2.3% Black, 11.6% Asian, 12.8% another race, 7.6% Hispanic, 47.7% sexual minority (i.e. bisexual, gay/lesbian, queer, or another non-heterosexual identity)], 30.2% used cigarettes and 54.1% used cannabis in the past 30 days. Participants used e-cigarettes 18.22 days of the past 30 on average, with 42.4% of participants reporting use on all 30 days. Overall, 39.5% used disposable devices, and 30.2% used tanks with refillable liquids. Additionally, 29.1% typically used e-liquids containing THC and 48.8% nicotine salt; e-liquid flavors most commonly used were fruit/dessert (73.1%) and menthol (47.4%). On average, participants reported greater self-reported quit confidence (mean core=6.38) than importance (mean score=4.84); 43.6% of participants reported a past-year e-cigarette quit attempt, 16.3% reported being ready to quit within the next 30 days, and 39.0% in the next 6 months (Table 1).

**Table 1:** Participant characteristics in a sample of US young adults who used e-cigarettes in the past 30 days, from the VAPES study in Fall 2022 (N=172)

*Characteristics*	*n (%)*
**MSA**	
Atlanta	28 (16.3)
Boston	27 (15.7)
Minneapolis	29 (16.9)
Oklahoma City	19 (11.0)
San Diego	13 (7.6)
Seattle	38 (22.1)
Other	18 (10.5)
**Age** (years), mean (SD)	26.95 (4.91)
**Sex**	
Male	72 (41.9)
Female	99 (57.6)
Sexual minority^a^	82 (47.7)
**Race**	
White	126 (73.3)
Black	4 (2.3)
Asian	20 (11.6)
Other	22 (12.8)
Hispanic	13 (7.6)
**Other substance use in past 30 days**	
Cigarettes	52 (30.2)
Cannabis	93 (54.1)
**E-cigarette use characteristics**	
Number of days used in past 30 days, mean (SD)	18.22 (12.26)
**Device type**	
Disposable	68 (39.5)
Replaceable prefilled cartridges	37 (21.5)
Tank refillable with liquids	52 (30.2)
Mod system	12 (7.0)
Use of e-liquids with THC^b^	50 (29.1)
Use of e-liquids with nicotine salt^b^	84 (48.8)
**Flavors most commonly used^c^**	
Tobacco	16 (9.4)
Menthol	81 (47.4)
Fruit/dessert	125 (73.1)
Other	27 (15.8)
**E-cigarette cessation-related factors**	
Importance of quittingd, mean (SD)	4.84 (3.31)
Confidence to quitd, mean (SD)	6.38 (3.14)
Past-year quit attempt	75 (43.6)
**Readiness to quit**	
Next 30 days	28 (16.3)
Next 6 months	67 (39.0)

a Sexual minority responses include gay/lesbian (n=12), bisexual (n=43), queer (n=9), or another non-heterosexual identity (n=18).

b Participants indicated how often they use e-liquids with THC and how often they use e-liquids with nicotine salt (never, rarely [coded as not using e-liquids with THC or nicotine salt, respectively], some of the time, most of the time, or all of the time [coded as using e-liquids with THC or nicotine salt, respectively]).

c Other flavors include: coffee or tea, alcoholic drink flavors, other. dScale: 0 = ‘not at all’ to 10 = ‘absolutely’.

The most frequently endorsed challenges to quitting or cutting down e-cigarette use were feeling too stressed, anxious, or down (41.9%), telling themselves they can quit/cut down later (35.5%), and using e-cigarettes out of boredom (25.6%). The most highly rated messages in terms of impact on motivation to quit e-cigarettes were related to quitting: to save money (mean core=4.78); to have better health, more money, and freedom (means core=4.15); and due to e-liquids containing toxic chemicals (means core=4.04). The most highly rated messages in terms of impact on confidence to quit e-cigarettes were related to patience (mean core=4.47), keeping busy (mean core=4.27), and asking others for help (mean core=3.84) (Table 2).

**Table 2:** Challenges to quitting/cutting down and perceived impact of intervention messaging targeting importance and confidence to quit in a sample of US young adults who used e-cigarettes in the past 30 days, from the VAPES study in Fall 2022 (N=172)

*Variables*	*n (%)*
**Challenges to quitting/cutting down^a^**	
I felt too stressed, anxious, or down	72 (41.9)
I told myself I could quit/cut down later	61 (35.5)
I was bored and turned to vaping	44 (25.6)
Public/social settings where smoking/vaping is allowed (‘is the norm’) made it a challenge to resist vaping	41 (23.8)
I had a hard time dealing with triggers or routines related to vaping	40 (23.3)
I struggled with withdrawal symptoms	39 (22.7)
I realized how addicted I was	38 (22.1)
I missed other enjoyable aspects of vaping	38 (22.1)
People important to me made it a challenge to resist vaping	29 (16.9)
I told myself vaping doesn’t pose any real health risks	26 (15.1)
I missed the flavors	25 (14.5)
I worried that I would use other tobacco products	24 (14.0)
I lost the motivation to quit or cut down after a slip-up	15 (8.7)
***Perceived impact of messages targeting motivation to quit^b^***	***Mean (SD)***
Did you know the average vaper spends $1000/year on vaping products? By quitting, you can save big and buy yourself something really nice.	4.78 (2.07)
Quitting vaping is hard, so enjoy all the things quitting vaping can give you, including healthier lungs, more money in your wallet, and freedom from addiction :)	4.15 (1.95)
Think vaping is harmless? Think again. E-juice contains many toxic chemicals, including chemicals found in weed killers and car exhausts.	4.04 (2.12)
Pods and e-liquids may contain very high levels of nicotine. This can harm your brain and affect your performance at work or school.	3.78 (2.08)
Did you know that >60% of young adults like you want to quit vaping?	3.21 (1.90)
Aggregated impact of messages on motivation to quit	3.99 (1.61)
**Perceived impact of messages targeting confidence to quit^c^**	
Remember that quitting is a process. Be patient with yourself and proud of all your hard work.	4.47 (1.99)
To beat cravings, keep your hands busy. Other vapers recommend mint toothpicks or lollipops.	4.27 (1.96)
It’s normal to feel irritable or out of sorts the first few days after quitting. Tell your friends, family, or roommate(s) that you’re quitting and ask for help if you need it.	3.84 (1.90)
Exercise is a great way to de-stress and deal with withdrawal. Try going for a short walk, run, or bike ride - even just for 15 mins. You’ll feel better!	3.74 (1.98)
Nicotine patches, gum, and lozenges give you low doses of nicotine without the other harmful chemicals in your vape. This reduces withdrawal symptoms.	3.71 (1.99)
You are what you think. Tell yourself, ‘I dontꞌ need nicotine or tobacco’.	3.13 (1.93)
You can quit even if your friends or roommates vape. Ask them not to vape around you or give you their vape, even if you really want it.	3.11 (1.92)
We know these are tough times. Instead of reaching for your vape, try other ways of dealing with stress or anxiety - like listening to some upbeat music.	2.86 (1.90)
Aggregated impact of messages on confidence to quit	3.63 (1.51)
***Recommendation for quitting e-cigarettes and cigarettes^d^***	***n (%)***
Quit both at the same time	39 (22.7)
Quit cigarettes first, then e-cigarettes	105 (61.0)
Quit e-cigarettes first, then cigarettes	9 (5.2)
**Recommendation for quitting e-cigarettes, cigarettes, and cannabis^e^**	
Quit all three at the same time	19 (11.0)
Quit cigarettes, then cannabis, then e-cigarettes	19 (11.0)
Quit cannabis, then cigarettes, then e-cigarettes	19 (11.0)
Quit cigarettes, then e-cigarettes, then cannabis	69 (40.1)
Quit e-cigarettes, then cigarettes, then cannabis	10 (5.8)

a Other, n=1; don’t know, n=5; none of the above, n=6; never tried to quit/reduce, n=27.

b Scale: 1 = ‘no impact at all’ to 7 = ‘a lot of impact’; Cronbach’s alpha=0.85.

c Cronbach’s alpha=0.91.

d Don’t know, n=17; other, n=2.

e Don’t know, n=24; other, n=12.

Multivariable linear regression indicated that perceiving greater impact of messages targeting motivation was associated with greater quit importance (B=1.16; 95% CI: 0.71–1.60, p<0.001); however, perceived impact of messages targeting confidence was not associated with quit importance. Additionally, neither the perceived impact of messages targeting motivation nor confidence was associated with perceived quiet confidence (Table 3). Older age (B= -0.12; 95% CI: -0.22–0.02, p=0.019) and more days of e-cigarette use (B= -0.09; 95% CI: -0.13 – -0.05, p<0.001) were associated with lower self-reported quit confidence. No other factors were significant in either model.

**Table 3:** Adjusted linear regression analyses examining the perceived impact of intervention messages in relation to motivation to quit e-cigarette use and confidence in quitting e-cigarette use in a sample of US young adults who used e-cigarettes in the past 30 days, from the VAPES study in Fall 2022

*Variable*	*Importance*			*Confidence*		
	*B*	*95% CI*	*p*	*B*	*95% CI*	*p*
**Age** (years)	-0.03	-0.13–0.07	0.601	-0.12	-0.22 – -0.02	0.019
**Female** (Ref. male)	-0.24	-1.23–0.76	0.639	-0.58	-1.57–0.41	0.249
**Race** (Ref. White)						
Black	-0.17	-3.27–2.93	0.913	1.14	-1.97–4.25	0.470
Asian	0.16	-1.32–1.63	0.834	-0.53	-2.01– 0.94	0.477
Other	-0.99	-2.36–0.39	0.158	-0.75	-2.12–0.62	0.283
Hispanic (Ref. non-Hispanic)	-0.54	-2.26–1.19	0.540	-0.13	-1.86–1.60	0.882
Sexual minority (Ref. heterosexual)	-0.72	-1.73–0.30	0.164	0.02	-1.00–1.03	0.973
**E-cigarette and other substance use**						
Number of days of e-cigarette use	0.03	-0.01–0.07	0.151	-0.09	-0.13 – -0.05	<0.001
Current cigarette use	-0.04	-1.03–0.96	0.941	-0.75	-1.74–0.25	0.139
Current cannabis use	-0.38	-1.31–0.54	0.411	-0.61	-1.53–0.31	0.193
**Perceived impact of messages**						
Targeting motivation	1.16	0.71–1.60	<0.001	0.15	-0.30–0.60	0.501
Targeting confidence	-0.25	-0.73–0.23	0.308	-0.09	-0.57–0.40	0.723
Adjusted R2		0.221			0.137	

Importance and confidence were assessed by asking participants to indicate the importance of quitting and their confidence in quitting cigarettes on a scale from 0 = ‘not at all’ to 10 = ‘absolutely’. Analyses were adjusted for sociodemographic and other substance use.

Polysubstance use status was not associated with recommendations for quitting e-cigarettes and cigarettes but was associated with recommendations for quitting e-cigarettes, cigarettes, and cannabis (Table 4). Exclusive e-cigarette users and e-cigarette-cigarette users (vs e-cigarette–cannabis users) were more likely to recommend first quitting cannabis, then cigarettes, and then e-cigarettes. E-cigarette–cannabis users (vs e-cigarette–cigarette users and poly-users of all three) were more likely to endorse quitting cigarettes, then e-cigarettes, then cannabis. Finally, poly-users of all three (vs exclusive e-cigarette users) were more likely to report quitting e-cigarettes, then cigarettes, then cannabis.

**Table 4:** Chi-squared analysis examining cigarette and cannabis poly-use status in relation to recommendations for quitting polysubstance use in a sample of US young adults who used e-cigarettes in the past 30 days, from the VAPES study in Fall 2022 (N=172)

*Recommendations*	*All*	*No polyuse with cannabis or cigarettes*	*Cannabis use only*	*Cigarette use only*	*Use of both cannabis and cigarettes*	
	*n (%)*	*n (%)*	*n (%)*	*n (%)*	*n (%)*	*p*
Total	172 (100)	59 (34.3)	61 (35.5)	20 (11.6)	32 (18.6)	
Quitting e-cigarettes and cigarettes						0.093
Quit both at the same time	39 (22.7)	10 (16.9)	13 (21.3)	4 (20.0)	12 (37.5)	
Quit cigarettes first, then e-cigarettes	105 (61.0)	40 (67.8)	40 (65.6)	10 (50.0)	15 (46.9)	
Quit e-cigarettes first, then cigarettes	9 (5.2)	1 (1.7)	2 (3.3)	2 (10.0)	4 (12.5)	
Don’t know	17 (9.9)	8 (13.6)	5 (8.2)	3 (15.0)	1 (3.1)	
Other	2 (1.2)	0 (0)	1 (1.6)	1 (5.0)	0 (0)	
**Quitting e-cigarettes, cigarettes, and cannabis**						**<0.001**
Quit all three at the same time	19 (11.0)	7 (11.9)	7 (11.5)	1 (5.0)	4 (12.5)	
Quit cigarettes, then cannabis, then e-cigarettes	19 (11.0)	11 (18.6)	5 (8.2)	2 (10.0)	1 (3.1)	
Quit cannabis, then cigarettes, then e-cigarettes	19 (11.0)	**10 (16.9)^a^**	**1 (1.6)^b^**	**5 (25.0)^a^**	**3 (9.4)^a,b^**	
Quit cigarettes, then e-cigarettes, then cannabis	69 (40.1)	**19 (32.2)^a^**	**35 (57.4)^b^**	**4 (20.0)^a^**	**11 (34.4)^a,b^**	
Quit e-cigarettes, then cigarettes, then cannabis	10 (5.8)	**0 (0)^a^**	**5 (8.2)^a,b^**	**0 (0)^a,b^**	**5 (15.6)^b^**	
Don’t know	24 (14.0)	**9 (15.3)^a,b,c^**	**6 (9.8)^c^**	**7 (35.0)^b^**	**2 (6.3)^a,c^**	
Other	12 (7.0)	3 (5.1)	2 (3.3)	1 (5.0)	6 (18.8)	

Values with different superscripts indicate significant post hoc differences at p<0.05.

## DISCUSSION

In this sample of young adult e-cigarette users, rates of poly-use with cigarettes (30.2%), cannabis (54.1%), and both cigarettes and cannabis (18.3%) were within 5% of 2021 national estimates for those aged 18–34 years^13^. Young adults in this study used e-cigarettes more frequently than recent national estimates (about 8%), with an average of about 18 days of use among those aged 18–34 years^14^. Rates of past-year e-cigarette quit attempts (43.6%) and readiness to quit (55.3% in next 6 months) were higher in the current study relative to previous studies (<30% past-year quit attempts^15,16^, <35% readiness to quit)^15^, potentially due to regulatory (e.g. FDA’s ban on unauthorized flavored cartridge-based e-cigarettes)^17^ and societal events (e.g. e-cigarette and vaping associated lung injury)^18^ that have increased awareness of e-cigarette-related harm and addiction^19^.

Multivariable regression findings indicated that those with a greater perceived impact of messages targeting motivation reported higher e-cigarette quitting importance. However, neither the perceived impact of messages targeting importance nor confidence were associated with participants’ self-reported quitting confidence, which may be due to participants’ high ratings of quit confidence (6.38 on a 10-point scale) relative to quit importance (4.84). Messages rated as most impactful on young adults’ motivation to quit e-cigarettes were related to saving money, better health, and the toxic chemicals in e-cigarettes, suggesting that messages targeting the health consequences and financial burden of regular e-cigarette use may be the most impactful in promoting the perceived importance of quitting among young adult e-cigarette users.

Young adults also varied in their recommendations for quitting e-cigarettes along with other products based on their poly-use profiles. Those who used e-cigarettes and cigarettes recommended quitting cannabis first, followed by cigarettes, then e-cigarettes. Conversely, those who used e-cigarettes and cannabis recommended quitting cigarettes first, followed by e-cigarettes, then cannabis. Users of all three products recommended quitting e-cigarettes first, followed by cigarettes, then cannabis, relative to e-cigarette-only users. Given high rates of e-cigarette use along with cannabis and/or cigarettes, cessation efforts targeting the use of multiple products should be tailored to the order in which young adults prefer to quit use based on their own use profiles.

### Limitations

Current findings should be interpreted in light of certain limitations. First, data were cross-sectional, and thus, directionality and causality cannot be inferred. Second, this study is limited in generalizability to other young adults in the included MSAs or across the US, given purposive sampling to recruit targeted populations of young adults using e-cigarettes and cigarettes and the use of social media recruitment. Third, these analyses are limited by the self-report nature of assessments, which are subject to response bias. Finally, data are under-representative of racial and ethnic minorities and do not include non-English speaking individuals. Future research should assess associations among study constructs using longitudinal data with a more generalizable sample.

## CONCLUSIONS

Findings underscore the importance of messages that promote the health and financial consequences of e-cigarettes in promoting perceived e-cigarette quitting importance among young adults who are not ready to quit. It remains essential to continue to monitor the associations observed in the current study. Findings expand upon previous research demonstrating the role of messages promoting e-cigarette cessation among young adults ready to quit only. Moreover, findings suggest that e-cigarette cessation interventions should account for young adults’ use of other products that occur alongside e-cigarette use (i.e. cigarettes, cannabis), as those with different poly-use profiles have different cessation preferences. Future research should test the impact of these messages on young adults’ motivation to quit and e-cigarette cessation-related outcomes.

## Data Availability

The data supporting this research are available from the authors upon reasonable request.
